# Seasonal variation and drought effects on colonization and community structure of arbuscular mycorrhizal fungi and dark septate endophytes in desert plants

**DOI:** 10.3389/fpls.2026.1849641

**Published:** 2026-09-04

**Authors:** Hengfang Wang, Li Sun, Yabei Zhang

**Affiliations:** 1 College of Ecology and Environment, Xinjiang University, Urumqi, Xinjiang, China; 2 Key Laboratory of Oasis Ecology of Education Ministry, Xinjiang University, Urumqi, Xinjiang, China; 3 Xinjiang Jinghe Observation and Research Station of Temperate Desert Ecosystem, Ministry of Education, Xinjiang University, Jinghe, China

**Keywords:** community composition, drought conditions, fungal colonization, host identity, seasonality

## Abstract

Arbuscular mycorrhizal fungi (AMF) and dark septate endophytes (DSE) are common root-associated fungal groups in natural ecosystems. However, how their colonization and community composition vary among seasons under natural drought conditions remains unclear. We investigated *Populus euphratica* and *Haloxylon ammodendron* in the Ebinur Lake Wetland National Nature Reserve, China. Microscopic observation and high-throughput sequencing were combined to characterize AMF and DSE colonization and community composition across spring, summer, and autumn under low, moderate, and high drought conditions. The results showed that drought strongly affected fungal colonization, with responses varying between host species. AMF colonization in *P. euphratica* increased to 90% under high drought conditions, and its response to drought differed among seasons, whereas DSE colonization responses were less season-dependent. AMF communities changed markedly across drought conditions, whereas DSE communities remained comparatively stable. Soil environmental variation was driven mainly by seasonality, and the colonization rates of both fungal groups were positively associated with soil ammonium nitrogen. Co-occurrence structure also varied seasonally, with the autumn network showing the greatest connectivity and complexity and a predominance of positive correlations. Overall, drought, seasonality, and host identity jointly shaped the colonization, community composition, and association patterns of root endophytic fungi in desert plants. These findings reveal contrasting responses of AMF and DSE to environmental stress and provide ecological insights relevant to dryland restoration.

## 1 Introduction

Endophytic fungi form symbiotic relationships with host plants, colonizing roots, stems, and leaves while enhancing growth and development (Hardoim et al., 2015; Ayala et al., 2023). Colonization of roots by beneficial fungi can promote root development and increase plant biomass, particularly under stressful conditions (Contreras-Cornejo et al., 2009; Li et al., 2019; Tang et al., 2024). These fungi enhance water-use efficiency by reducing root water loss—a critical adaptation in water-limited arid ecosystems (He et al., 2019; Willing et al., 2024). Diverse root-associated endophytic fungi, including arbuscular mycorrhizal fungi within Glomeromycotina and fine root endophytes within Mucoromycotina, can co-colonize terrestrial plant roots and contribute to plant health by promoting growth, nutrient uptake, and stress tolerance (Bonfante and Venice, 2020; Trivedi et al., 2020; Shi et al., 2023; Prout et al., 2024). In addition to enhancing nutrient and water uptake and increasing drought resistance (Kuldau and Bacon, 2008; Bueno de Mesquita et al., 2018; Trivedi et al., 2020), root-associated endophytic fungi can shape rhizosphere microbial assembly and nutrient cycling, thereby acting as a functional bridge between plant root adaptation and soil ecological processes under environmental stress (Sebastiana et al., 2019; Zhou et al., 2025).

Arbuscular mycorrhizal fungi (AMF) colonize plant roots and establish mutualistic symbioses that are essential to terrestrial ecosystems (Brachmann and Parniske, 2006). Taxonomically, most AMF are affiliated with Glomeromycotina, although recent studies have shown that some fine root endophyte AM morphotypes belong to Mucoromycotina, indicating that AM symbioses can involve distinct fungal lineages (Redecker et al., 2013; Spatafora et al., 2016; Seeliger et al., 2024). A defining feature of this symbiosis is the formation of arbuscular structures within root cortical cells, which facilitate nutrient exchange between host plants and fungal partners (Powell and Rillig, 2018; Mahmoudi et al., 2020). Through these associations, AMF enhance mineral nutrient acquisition, facilitate soil organic matter formation and stabilization, support plant community establishment through common mycorrhizal networks, and contribute to ecosystem stability, particularly in arid environments (Smith and Read, 2008; Wu et al., 2023; Willing et al., 2024; Zhou et al., 2025). Understanding how AMF respond to seasonal variation and drought stress is therefore critical for elucidating plant-fungal adaptation strategies in arid ecosystems.

Dark septate endophytes (DSE) are functionally important fungal symbionts that colonize plant roots via inter- and intracellular pathways (Rodriguez et al., 2009; de Oliveira and Pereira, 2025). As ubiquitous root colonizers (Mandyam and Jumpponen, 2005), DSE are morphologically defined by melanized hyphae with regular septation (Della Monica et al., 2015). DSE demonstrate broad ecological adaptability, colonizing diverse habitats from temperate to extreme environments and conferring tolerance to multiple abiotic stresses such as heavy metals, drought, and salinity (Xie et al., 2024b; Lu et al., 2025). Mechanistically, DSE enhance host stress resilience by modifying root architecture, particularly through stimulating lateral root formation (Li et al., 2019), thereby improving belowground biomass and root length under stress. Furthermore, DSE mycelial networks substantially expand the absorptive surface area for water and nutrient acquisition while modulating soil nutrient cycling under stress conditions (Barrow, 2003; He et al., 2019).

AMF and DSE are among the most abundant endophytic communities (Scervino et al., 2009; Netherway et al., 2024), exhibiting dynamic colonization patterns that vary with plant developmental stages, seasons, and habitats (Li et al., 2018). These fungi frequently co-colonize root zones (Claassens et al., 2018) and can synergistically enhance plant growth (Xie et al., 2021; Bi et al., 2024), despite occupying distinct ecological niches. AMF predominantly occur in soils with lower C/N ratios and higher pH, whereas DSE favor environments with lower pH and elevated organic matter content (Huusko et al., 2016). However, DSE associated with AMF hyphae may disrupt carbon and nutrient exchange in AMF-plant symbioses (Deveautour et al., 2021), potentially reducing symbiotic efficiency (Giesemann et al., 2021). The coexistence mechanisms between AMF and DSE, including competitive, independent, and cooperative interactions, remain poorly understood. Therefore, the net effects of these interactions on ecological restoration require further investigation (Bi and Xie, 2021).

Given ecosystem-level variability in nutrient availability, elucidating how endophytic fungal colonization strategies and plant-fungal relationships vary across environmental gradients is critical (Huo et al., 2021). In arid and semi-arid ecosystems, soil moisture represents the primary determinant of endophytic fungal colonization dynamics (Ayala et al., 2023). The extensive root systems of desert plants such as *P. euphratica* and *H. ammodendron* facilitate survival in desert environments while creating microhabitats for rhizosphere fungi (Yao et al., 2020). Thus, characterizing the seasonal dynamics of endophytic fungal communities in desert plants and their drought adaptation mechanisms under natural conditions is imperative.

The soils across Ebinur Lake ecosystems are uniformly saline-sandy in texture (Gong et al., 2019; Wang et al., 2019; Chen et al., 2025). The basin’s desert ecosystem contains distinct vegetation formations dominated by *P. euphratica*, *H. ammodendron*, and *Tamarix ramosissima* (Kong and Nurbayi, 2008). While the region’s floristic diversity has been well documented, understanding of mycorrhizal fungi-native plant interactions in desert ecosystems remains limited. Existing research in desert ecosystems has primarily examined AMF diversity and its correlation with plant diversity under drought (Wei, 2015; Lv et al., 2016), while neglecting DSE. To address this gap, we investigated AMF and DSE colonization in *P. euphratica* and *H. ammodendron* roots across seasons in Ebinur Lake’s arid desert region. We address two key questions: (1) How do seasonal and drought conditions influence AMF and DSE colonization rates and diversity, and are these patterns mediated by host identity and soil physical and chemical factors? (2) Do drought and seasonality drive AMF/DSE community restructuring, exhibiting host specificity, genus replacement, and seasonal co-occurrence patterns?

## 2 Materials and methods

### 2.1 Study site

**Figure 1:**
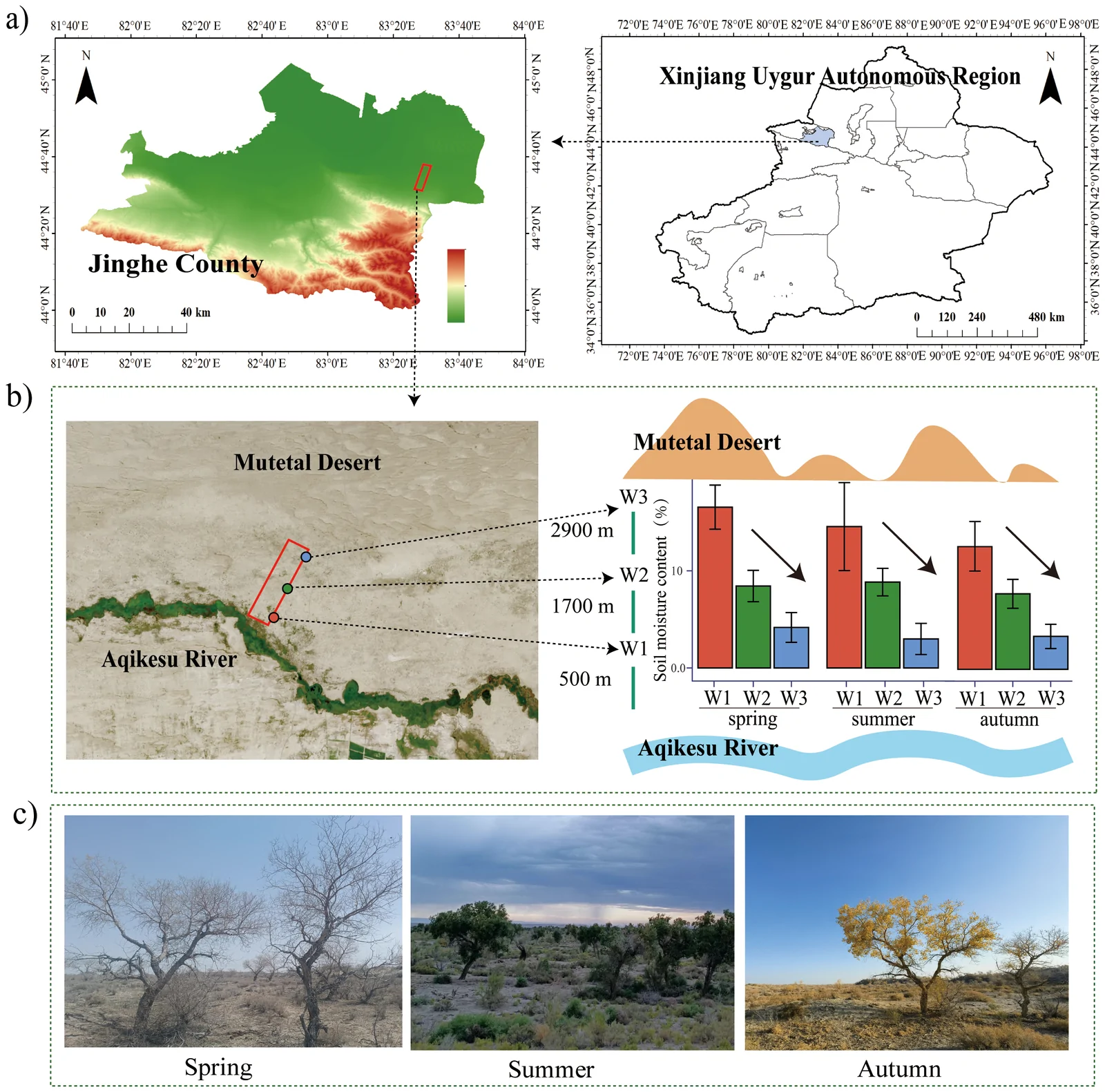
Study area location, sampling sites, and representative seasonal landscapes under different drought conditions. **(a)** Geographic location of the study area. **(b)** Sampling sites and soil moisture under different drought conditions. **(c)** Representative landscapes across seasons. W1, W2, and W3 denote low, moderate, and high drought conditions, respectively.

The study area is located in the Ebinur Lake Wetland National Nature Reserve, Jinghe County, Xinjiang Uygur Autonomous Region, China (44°30′–45°09′ N, 82°36′–83°50′ E; **Figure 1a**). The reserve covers a total area of approximately 2,670.85 km^2^ and is characterized by a typical temperate continental arid climate. The region receives extremely low annual precipitation and experiences high evaporation rates, with uneven precipitation distribution and intense evaporative demand. The mean annual precipitation is less than 150 mm, whereas the potential annual evaporation exceeds 1,500 mm. The mean annual temperature is 9.1 °C (Zhang et al., 2016; Chen et al., 2025). The dominant soil types in the study area include gray desert soil, aeolian sandy soil, and gray-brown desert soil (Wang et al., 2019). The vegetation in this region exhibits transitional characteristics between the eastern margin of the Central Asian desert and the western margin of the Mongolian Plateau desert steppe, and it harbors relatively high biodiversity (Li et al., 2022; Chen et al., 2025). The main dominant plant species include *P. euphratica*, *H. ammodendron*, *Tamarix chinensis*, *Halostachys caspica*, *Kalidium foliatum*, *Reaumuria songonica*, and *Alhagi sparsifolia* (Yang et al., 2022; Wang et al., 2019).

The sampling sites of this study were located in a typical desert ecosystem near the Dongdaqiao Management Station within the reserve (**Figure 1b**). Due to the scarcity of precipitation in this region, river recharge serves as the primary source for maintaining soil moisture and groundwater levels. Previous studies have shown that with increasing distance from the river, groundwater depth gradually increases and soil moisture content significantly decreases, forming a stable soil moisture gradient (Yang et al., 2022). In addition, as the distance from the riverbank increases, soil organic matter content and water-holding capacity progressively decline (Wang et al., 2019). In arid desert ecosystems, changes in overall soil bulk density and sand content further increase the soil wilting point and reduce water supply capacity (Jiang et al., 2022). The combined effects of these factors lead to a gradual decline in soil moisture along the spatial gradient, thereby intensifying the impact of drought stress on plant roots and their rhizosphere microbial communities (Gong et al., 2019; Hu et al., 2021). Soils in the study area are significantly affected by drought, and soil moisture, organic carbon, nutrient content, and enzyme activity decrease markedly with increasing soil moisture gradient intensity (Huang et al., 2024; Chen et al., 2025). Based on this stable soil moisture gradient, sampling transects were established perpendicular to the riverbank. Three representative sites were selected according to their distance from the river and were designated as W1, W2, and W3. These sites were located approximately 500 m, 1,700 m, and 2,900 m from the riverbank, respectively, representing progressively increasing drought conditions from near to far (**Figure 1b**). Accordingly, W1, W2, and W3 were defined as low, moderate, and high drought conditions, respectively. Plants and their rhizosphere microbial communities distributed along this gradient experience progressively stronger drought stress (Gong et al., 2019; Li et al., 2022).

### 2.2 Sample collection

Under the three drought conditions (W1, W2, and W3), three independent 30 × 30 m parallel plots were established for each condition, resulting in a total of nine plots. Each plot was treated as a statistical replicate (n = 3). *P. euphratica* and *H. ammodendron* were stably distributed within each plot. These two species are widely distributed in the study area and exhibit strong ecological representativeness (Li et al., 2022; Mo et al., 2025). Samples were collected in spring (May), summer (August), and autumn (October) (**Figure 1c**). Each sampling was conducted within fixed plots. In each plot, three individuals of each of the two plant species were randomly selected. The selected individuals were healthy, free from disease and mechanical damage, and exhibited similar growth status. For each individual, root samples were collected from the fine root-dense zone within the 0–20 cm soil layer surrounding the trunk (Huang and Guo, 2009; Xia et al., 2015). Although both species are deep-rooted desert phreatophytes, this shallow soil layer was selected because fine absorptive roots and root-associated mycorrhizal colonization are generally concentrated and more active in the upper root-zone soil (Jackson et al., 1996; Wang et al., 2024b). Fine root samples from the three individuals within the same plot were pooled to form one composite sample per plot. This procedure was implemented to reduce the influence of microscale spatial heterogeneity and individual variation on the characterization of root-associated fungal communities. Similar plot-level composite sampling approaches have been widely adopted in rhizosphere and soil microbial studies (Smalla et al., 2001).

During sample collection, surface vegetation and debris were first removed. Fine roots were then carefully excavated and located using a small shovel, and root samples were collected. The collected root samples were immediately labeled, sealed in sterile bags, and transported to the laboratory under low-temperature conditions. Each composite sample was further divided into two fine root subsamples. One subsample was stored at 4 °C for the determination of mycorrhizal colonization rates, and the other was stored at −80 °C for subsequent DNA extraction (Zheng et al., 2018). Rhizosphere soil was obtained by gently shaking the roots and washing them with phosphate-buffered saline (PBS). The tightly adhering soil particles on the root surface were subsequently collected by centrifugation (Edwards et al., 2015). The collected rhizosphere soil samples were placed in labeled self-sealing bags and transported to the laboratory for further analysis.

### 2.3 Determining the soil physical and chemical properties

Fresh soil samples were transported to the laboratory in pre-weighed aluminum boxes, weighed, oven-dried at 105 °C for 24 h to constant mass, and reweighed to calculate gravimetric soil water content. The samples were then air-dried and sieved to obtain root-zone soil samples, which were analyzed for their physicochemical properties (**Supplementary Table 1**). These properties included soil water content (SW), soil salinity (SC), soil pH, soil organic carbon (SOC) content, total phosphorus (TP), total nitrogen (TN), available phosphorus (AP), ammonium nitrogen (AN), nitrate nitrogen (NN), urease (Ure), and alkaline phosphatase (ALP) (Bao, 2000).

### 2.4 Methods for quantifying AMF and DSE colonization in plant roots

**Figure 2:**
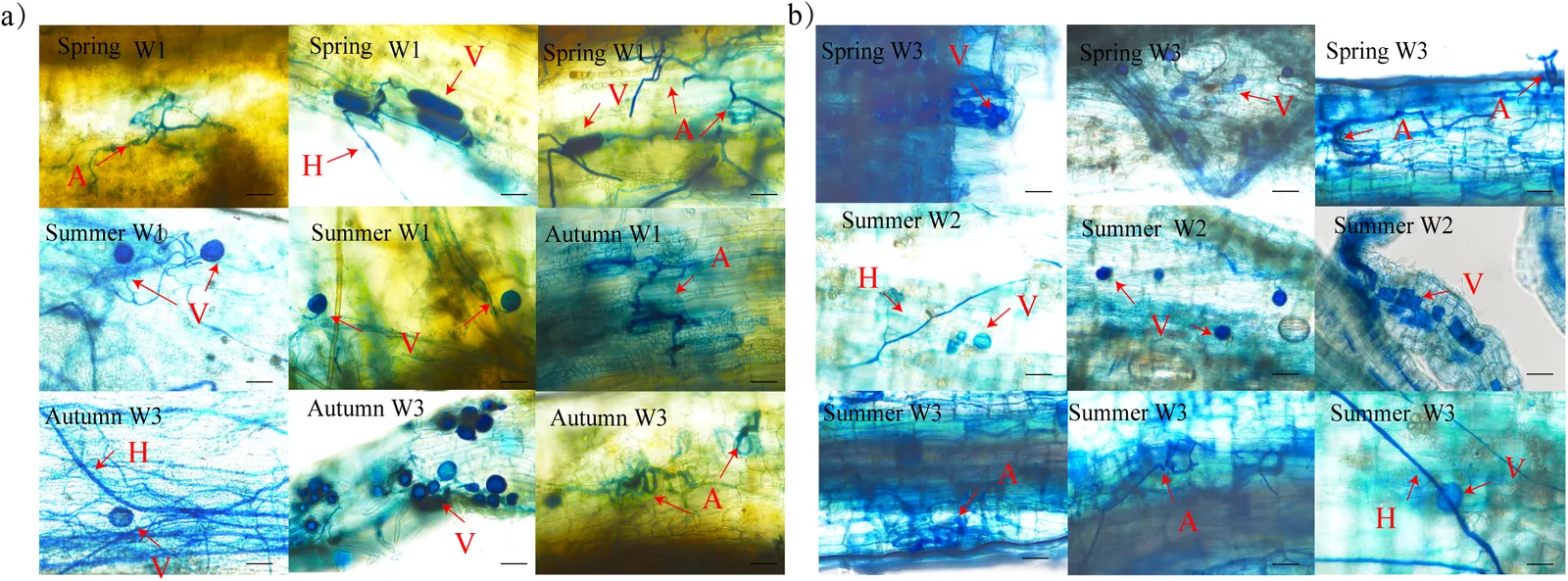
Typical morphological structures of AMF in the roots of *P. euphratica* and *H. ammodendron*. Scale bars = 50 μm. **(a)** *P. euphratica*; **(b)** *H. ammodendron*. W1, low drought condition; W2, moderate drought condition; W3, high drought condition. H, AMF hypha; A, arbuscule; V, vesicle.

**Figure 3:**
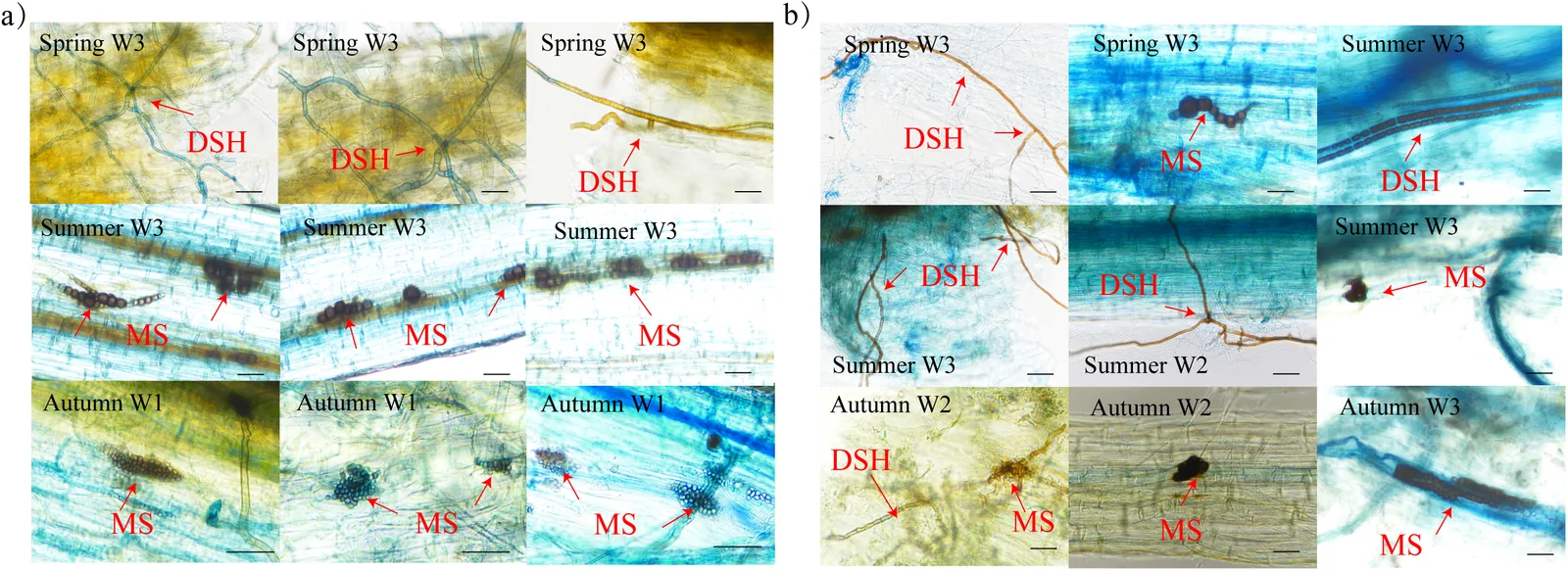
Typical morphological structures of DSE in the roots of *P. euphratica* and *H. ammodendron*. Scale bars = 50 μm. **(a)** *P. euphratica*; **(b)** *H. ammodendron*. DSH, DSE hypha; MS, microsclerotia.

Sufficiently fine roots were randomly selected from the root samples. The fine roots were rinsed with distilled water to remove soil particles. They were then cut into segments approximately 1 cm in length and placed in test tubes. To stain the roots, we applied the Phillips and Hayman staining method, which involved soaking the roots in a 10% KOH solution and incubating them at 90 °C until cleared and bleached (10–40 min, with iterative observation until the roots became transparent). After rinsing with distilled water, the roots were acidified with a 2% HCl solution for 5–10 minutes and washed again. Finally, the roots were stained with a 0.05% trypan blue solution at 90 °C for 90 minutes and decolorized in lactic acid glycerol for 120 minutes (Phillips and Hayman, 1970). The structures of AMF and DSE were observed under a research-grade inverted microscope (Nikon ECLIPSE Ti2, Japan), and typical structures were photographed and saved. The hyphae, arbuscules, and vesicles of AM fungi, all stained blue by the dye, were counted separately (**Figure 2**). In contrast, the dark septate hyphae and clusters of swollen, round, thick-walled cells (microsclerotia), characteristic of DSE, were generally dark brown and recorded as DSE structures (**Figure 3**) (Gallaud, 1904; Jumpponen and Trappe, 1998). Stained roots were observed under a microscope at 200× magnification. For each sample, 30 root segments (1 cm in length) were randomly selected and evenly arranged on three glass slides for microscopic examination. At least 150 intersections were recorded per slide. Mycorrhizal colonization rates were determined using the gridline intersection method (McGonigle et al., 1990). At each intersection, the type of mycorrhizal colonization was recorded, including hyphae, vesicles, arbuscules, and their various combinations. In addition, within each microscopic field, the presence of non-colonized roots, vesicles, arbuscules, AMF hyphae, DSE hyphae, and microsclerotia was recorded. Representative structures were documented by microphotography.

### 2.5 DNA extraction, amplification, and ITS amplicon sequencing

The frozen fine-root samples were sent to Shanghai Majorbio Bio-pharm Technology Co., Ltd. (Shanghai, China) for ITS amplicon sequencing with spike-in-based absolute quantification. Total DNA was extracted from the root samples using the E.Z.N.A.^®^ Soil DNA Kit (Omega Bio-tek, Norcross, GA, USA) according to the manufacturer’s instructions. The quality of the extracted DNA was assessed using 1% agarose gel electrophoresis, and its concentration and purity were measured using a NanoDrop2000 (Thermo Scientific, USA). Using the extracted DNA as the template, the ITS1 region was amplified using the barcoded forward primer ITS1F (5′-CTTGGTCATTTAGAGGAAGTAA-3′) and the reverse primer ITS2R (5′-GCTGCGTTCTTCATCGATGC-3′) (Gardes and Bruns, 1993; Yurkov et al., 2020; Wang et al., 2024a). Sequencing was conducted using an Illumina NextSeq2000 platform (Shanghai Majorbio Bio-pharm Technology Co., Ltd.).

Raw paired-end sequencing reads were first subjected to quality control using fastp v0.19.6 (https://github.com/OpenGene/fastp) (Chen et al., 2018). High-quality reads were then merged using FLASH v1.2.11 (https://ccb.jhu.edu/software/FLASH/) (Magoč and Salzberg, 2011). During quality filtering, sequences were trimmed using Q20 as the quality threshold. Only high-quality sequences longer than 50 bp and without ambiguous bases (N) were retained. Paired-end reads were subsequently merged with a minimum overlap of 10 bp and a maximum mismatch ratio of 0.2. Finally, samples were demultiplexed based on barcode and primer sequences.

Sequences were clustered into operational taxonomic units (OTUs) at a 97% similarity threshold using UPARSE v7.1 (http://drive5.com/uparse/) (Edgar, 2013), and chimeric sequences were removed during the clustering process. This OTU-based clustering approach is a classical and widely recognized analytical strategy in fungal community studies. It remains reliable and robust for revealing the overall ecological response patterns of fungal communities along environmental gradients (Cline et al., 2017; Rzehak et al., 2024). Taxonomic classification was conducted using the RDP classifier (version 2.11; https://github.com/rdpstaff/classifier) against the UNITE 9.0 database, applying a confidence threshold of 70%. Spike-in OTUs were identified as internal standards, and a calibration curve was established based on the number of Spike-in DNA sequences recovered from each sample to estimate the absolute copy numbers of fungal taxa. Because Spike-in DNA was artificially added, Spike-in sequences were removed before downstream biological community analyses. Subsequent analyses were conducted using the Spike-in-filtered OTU table, which contained taxonomic assignments and abundance information for biological fungal taxa.

We acknowledge that the ITS region has limited taxonomic resolution and high sequence variability for Glomeromycota, and that universal fungal ITS primers may underestimate AM fungal diversity due to their relatively low amplification efficiency for this group (Öpik et al., 2013; Perez-Lamarque et al., 2022; Guillen-Otero et al., 2023). Universal ITS primers were used to profile AMF and DSE communities within the same root-associated fungal dataset, but AMF-specific 18S or LSU rRNA primers were not applied in parallel; this represents a methodological limitation. Accordingly, AM fungal community analyses were restricted to ITS-amplified OTUs assigned to Glomeromycota at the 97% OTU threshold (Tedersoo et al., 2022; Florence et al., 2025), and the results should not be interpreted as a complete or species-resolved characterization. Raw data were deposited in the NCBI Sequence Read Archive database under accession number PRJNA1046269.

### 2.6 Statistical methods

To examine the effects of season, plant species, drought conditions, and their interactions on AMF and DSE colonization rates, a three-way analysis of variance (three-way ANOVA) was conducted using the R package rstatix (v0.7.3; Kassambara, 2025). Prior to the ANOVA, the Shapiro-Wilk test for normality and Levene’s test for homogeneity of variances were performed using the `shapiro_test()` and `levene_test()` functions in the rstatix package, respectively. As some data did not meet the assumptions of normality and homogeneity of variances, the Kruskal-Wallis nonparametric test was further applied as a supplementary analysis to assess differences in colonization rates among treatment groups.

To evaluate the effects of drought conditions on fungal community composition, Bray-Curtis dissimilarities among samples were calculated from the OTU absolute abundance matrix obtained from sequencing analysis using the R package vegan (v2.7-3; Oksanen et al., 2026). Principal coordinates analysis (PCoA) was then performed to visualize differences in community structure under different drought conditions. Subsequently, based on the Bray-Curtis distance matrix, permutational multivariate analysis of variance (PERMANOVA) was conducted using the `adonis2()` function with 999 permutations to quantitatively assess the explanatory power and statistical significance of drought conditions on differences in community structure. Similarity percentage (SIMPER) analysis was further performed to identify the main taxa contributing to community differences under different drought conditions, and the contribution rate of each taxon to the overall community dissimilarity was calculated. In addition, indicator species analysis (IndVal.g method, number of permutations = 9,999) was conducted to identify indicator species associated with *P. euphratica* and *H. ammodendron*. Taxa with relatively high contribution rates were visualized. Pairwise comparisons of community composition among different seasons were performed to identify key taxa driving community differences during seasonal transitions.

Sankey diagrams illustrating the effects of season and drought conditions on AMF and DSE community composition were generated using relative abundance data with the R package ggalluvial (v0.12.6; Brunson and Read, 2026), to intuitively show proportional changes in dominant taxa across seasons, plant species, and drought conditions. To further elucidate seasonal differences in the responses of AMF and DSE communities to drought conditions, data from spring, summer, and autumn were analyzed separately using the corresponding calibrated OTU absolute abundance matrices. Based on the Bray-Curtis distance matrix, non-metric multidimensional scaling (NMDS) was performed to ordinate and visualize fungal community structure. The stress value was calculated using the `metaMDS()` function in the vegan package to evaluate the reliability of the ordination results. In addition, NMDS axis scores were extracted, and pairwise comparisons among the three drought conditions were performed using the Wilcoxon rank-sum test to assess differences in their distributions along the ordination axes. Figures were generated using the R package ggplot2 and integrated into a composite visualization comprising the main NMDS plot and boxplots on both sides, thereby intuitively illustrating the effects of drought conditions on AMF and DSE community structure.

To characterize soil environmental heterogeneity, principal component analysis (PCA) was conducted on standardized environmental variables using the prcomp() function. The resulting ordination was visualized in PC1-PC2 space with 95% confidence ellipses (ggplot2 v3.4.0; stat_ellipse()), stratified by season and plant species. A permutational multivariate analysis of variance was performed using the adonis2() function with 999 permutations to quantify the explanatory power (R^2^) and statistical significance (p-value) of season and plant species effects on overall environmental variability, based on Euclidean distance matrices. Concurrently, environmental factor vectors were projected onto PCA space via the envfit() function to identify key driving variables. Linear regression analysis was performed between PC1 and soil water content to determine the coefficient of determination and significance level. To explore the relationships between AMF and DSE colonization rates and environmental factors, Mantel tests were conducted to assess correlations. The Mantel test was performed using the `mantel_test()` function, and the results were visualized as correlation networks using the R package linkET (v0.1.0; Huang, 2021) to distinguish positive and negative correlations and their significance levels. In addition, Spearman correlation analysis was applied to calculate correlation coefficients between dominant genera and soil environmental factors. The false discovery rate (FDR) method was used to correct for multiple testing. Correlation results were visualized as heatmaps using the R package pheatmap (v1.0.13; Kolde, 2025). Bidirectional hierarchical clustering was performed for both fungal genera and soil factors to identify potential correlation patterns. To characterize the co-occurrence patterns and association structure among key AMF and DSE taxa, co-occurrence networks were constructed based on taxon abundance data using Spearman’s rank correlation analysis. To minimize spurious associations and improve network robustness, only strong and statistically significant correlations were retained as network edges, using thresholds of Spearman’s |r| ≥ 0.6 and Benjamini-Hochberg FDR-adjusted *p* < 0.05. Undirected networks were generated using the R package igraph (v2.2.2; Csárdi et al., 2026) according to node and edge data, and isolated nodes were removed to optimize network structure. Subsequently, multiple network topological metrics were calculated, including the number of nodes, number of edges, average degree, global clustering coefficient, network density, and the proportion of positive and negative correlations, to quantify network complexity and taxon association patterns. Network visualization was performed using the R package ggraph (v2.2.2; Pedersen, 2025), and nodes were arranged based on the Fruchterman-Reingold layout algorithm.

## 3 Results

### 3.1 Differential responses of AMF and DSE colonization rates to season, plant species, and drought conditions

Root staining and microscopic observations revealed that both AMF and DSE successfully colonized the roots of *P. euphratica* and *H. ammodendron*. Specifically, AMF developed characteristic structures within root tissues, including vesicles, arbuscules, and hyphal networks (**Figure 2**). DSE formed intercellular melanized septate hyphae and microsclerotia with varying pigmentation intensity, primarily localized in the epidermal layer and vascular bundle tissues of both plant species (**Figure 3**).

The colonization rates of AMF and DSE were systematically compared across seasons, plant species, and drought conditions. Analysis of variance showed that the main effects of plant species and drought conditions significantly influenced the colonization rates of both fungal groups (*p* < 0.001), whereas the main effect of season was not significant. Significant plant species × drought condition interactions were observed for both AMF and DSE colonization rates (AMF: *p* < 0.001; DSE: *p* = 0.001), indicating that the responses of the two host plants to drought conditions differed. Notably, AMF colonization rates showed significant season × drought interactions (*p* = 0.033) and marginally significant three-way interactions (*p* = 0.075), while DSE colonization exhibited no significant responses in these interactions (**Table 1**). These results suggest that AMF may have a more complex environmental response compared to DSE.

**Table 1:** Three-way ANOVA results for AMF and DSE colonization rates.

Source of Variation	AMF Colonization			DSE Colonization		
	df	F	*p*	df	F	*p*
Main Effects						
Season	2	0.865	0.429	2	1.235	0.303
Species	1	42.571	< 0.001***	1	67.032	< 0.001***
Drought conditions	2	29.071	< 0.001***	2	12.799	0.001**
Interaction Effects						
Season × Species	2	1.399	0.260	2	0.365	0.697
Season × Drought conditions	2	3.740	0.033*	2	1.732	0.191
Species × Drought conditions	1	23.315	< 0.001***	1	12.606	0.001**
Season × Species × Drought conditions	2	2.780	0.075	2	1.843	0.173

***p < 0.001; **p < 0.01; *p < 0.05.

**Figure 4:**
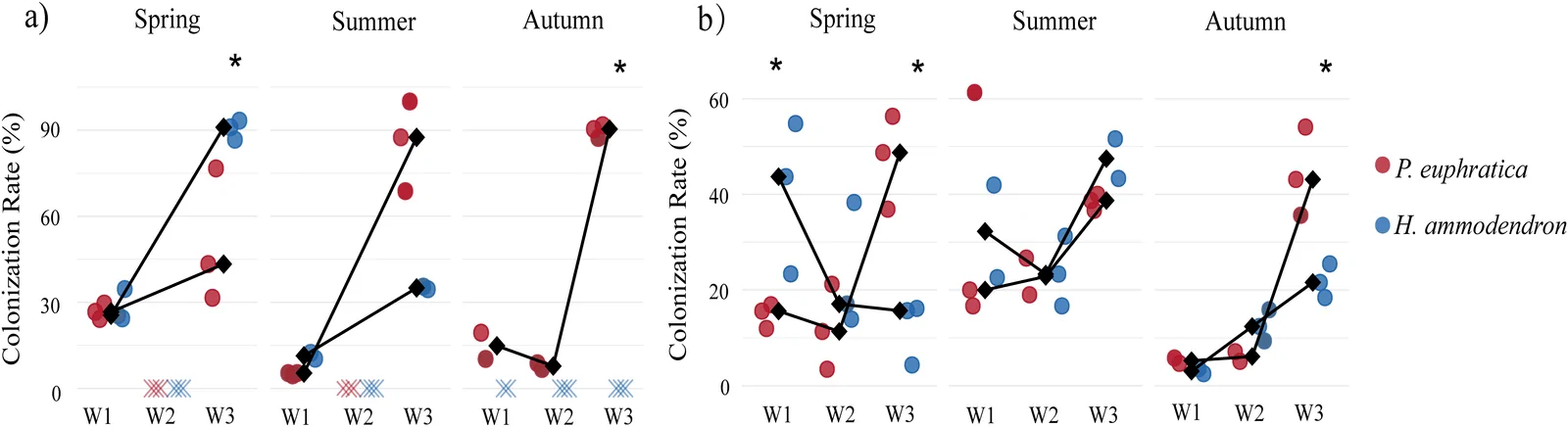
Seasonal variations in fungal colonization rates under drought conditions. **(a)** Variations in arbuscular mycorrhizal fungal colonization rates across drought conditions. **(b)** Variations in dark septate endophyte colonization rates across drought conditions. Red and blue dots represent *P. euphratica* and *H. ammodendron*, respectively. Solid black lines connect the mean colonization rates across treatments. In some samples, no AMF colonization was detected (colonization rate = 0). ***p < 0.001; **p < 0.01; *p < 0.05.

**Figure 5:**
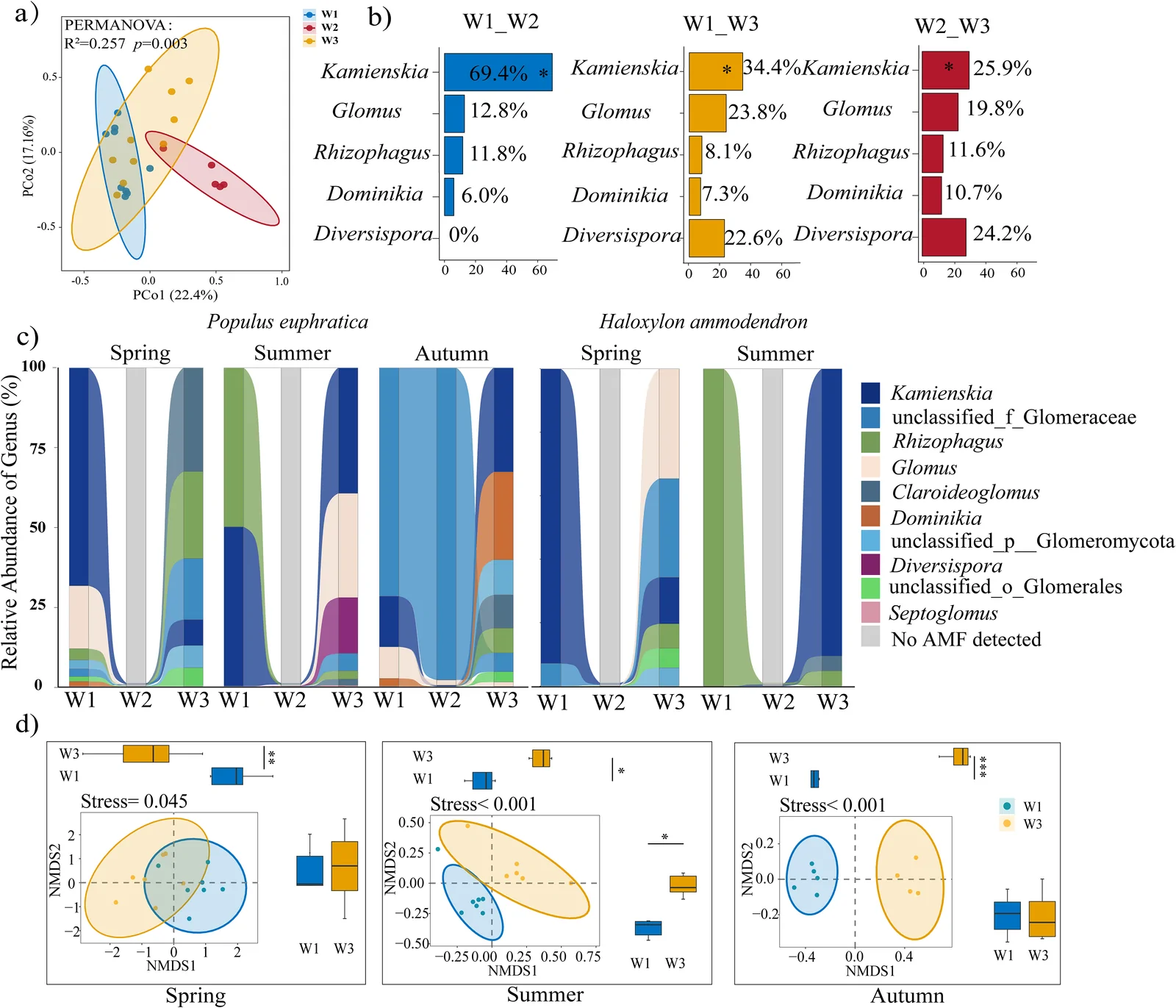
AMF community composition in the roots of *P. euphratica* and *H. ammodendron* under seasonal variation and different drought conditions. **(a)** Differences in AMF community composition among drought conditions based on Bray-Curtis dissimilarities calculated from the OTU absolute abundance matrix. **(b)** Major taxa contributing to AMF community dissimilarity based on SIMPER analysis. **(c)** Relative abundance-based AMF community composition across seasons, plant species, and drought conditions, showing proportional changes in dominant taxa. Gray bars indicate samples in which no AMF taxa were detected and do not represent missing data. **(d)** Differences in AMF community composition between W1 and W3 within each season based on the OTU absolute abundance matrix. W2 was excluded from the season-specific NMDS comparisons because detectable AMF community profiles were not consistently available across season × host combinations. W1, W2, and W3 represent low, moderate, and high drought conditions, respectively. ***p < 0.001; **p < 0.01; *p < 0.05.

Fungal colonization rates showed significant differences under various drought conditions (*p* < 0.05). Specifically, AMF colonization in *P. euphratica* demonstrated clear water dependence (**Figure 4a**), with colonization rates below 30% under W1 but significantly increasing to 90% under W3. This trend was particularly significant in spring and autumn (*p* < 0.05). However, under the W2 condition in spring and summer, arbuscules were not detected in the roots of *P. euphratica*. In addition, no AMF colonization was observed in *H. ammodendron* samples collected in autumn. Consistently, taxonomic composition analyses also indicated that no AMF-related taxa were detected in these samples (**Figures 4a**, **5c**).

In contrast, DSE exhibited more complex colonization dynamics (**Figure 4b**). During spring, *H. ammodendron* and *P. euphratica* displayed opposing responses of DSE colonization to drought conditions, yet both species reached their minimum values at the W2 level. During summer and autumn, however, both host plants showed similar response patterns: colonization rates were consistently lowest at W2 and highest at W3, with the autumn increase being statistically significant (*p* < 0.05).

### 3.2 Differences in AMF and DSE community composition across seasons and drought conditions

In this study, high-throughput sequencing was used to analyze AMF communities at the genus level under different seasonal and drought conditions. A total of 1 phylum, 1 class, 2 orders, 3 families, and 7 genera were identified (**Supplementary Table 2**). The DSE community comprised 1 phylum, 4 classes, 9 orders, 17 families, and 20 genera (**Supplementary Table 3**). Principal coordinates analysis (PCoA) based on Bray-Curtis dissimilarities revealed clear differences in AMF community composition among drought conditions. PERMANOVA further confirmed that drought intensity had a significant effect on AMF community structure (R^2^ = 0.257, *p* = 0.003; **Figure 5a**). SIMPER analysis indicated that community differences were primarily driven by the genera *Kamienskia*, *Glomus*, *Rhizophagus*, and *Diversispora*. Among them, *Kamienskia* contributed the most, accounting for 69.4%, 34.4%, and 25.9% of the dissimilarity in the W1–W2, W1–W3, and W2–W3 comparisons, respectively (**Figure 5b**). In spring, as drought intensity increased from W1 to W3, the relative abundance of *Kamienskia* gradually decreased, whereas the abundances of other genera increased, resulting in a more diversified community structure under the W3 condition (**Figure 5c**). In autumn, the number of detected AMF taxa in the roots of *P. euphratica* increased, particularly under the W3 condition. The number of taxa under the W3 condition was markedly higher than that under W1 and W2, and the dominant taxa shifted from unclassified groups to *Kamienskia* and *Dominikia* (**Figure 5c**). The NMDS ordination showed partial overlap between W1 and W3 in spring, although the difference remained significant (*p* < 0.01). Separation between W1 and W3 was more pronounced in summer (*p* < 0.05) and autumn (*p* < 0.001) (**Figure 5d**). Indicator species analysis (IndVal) revealed significant seasonal differences in *Rhizophagus* and *Dominikia* (*p* < 0.05) (**Supplementary Figure 1**). In addition, *Glomus*, *Septoglomus*, *Dominikia*, and *Diversispora* were detected exclusively in *P. euphratica* and were not observed in *H. ammodendron* (**Supplementary Table 4**).

**Figure 6:**
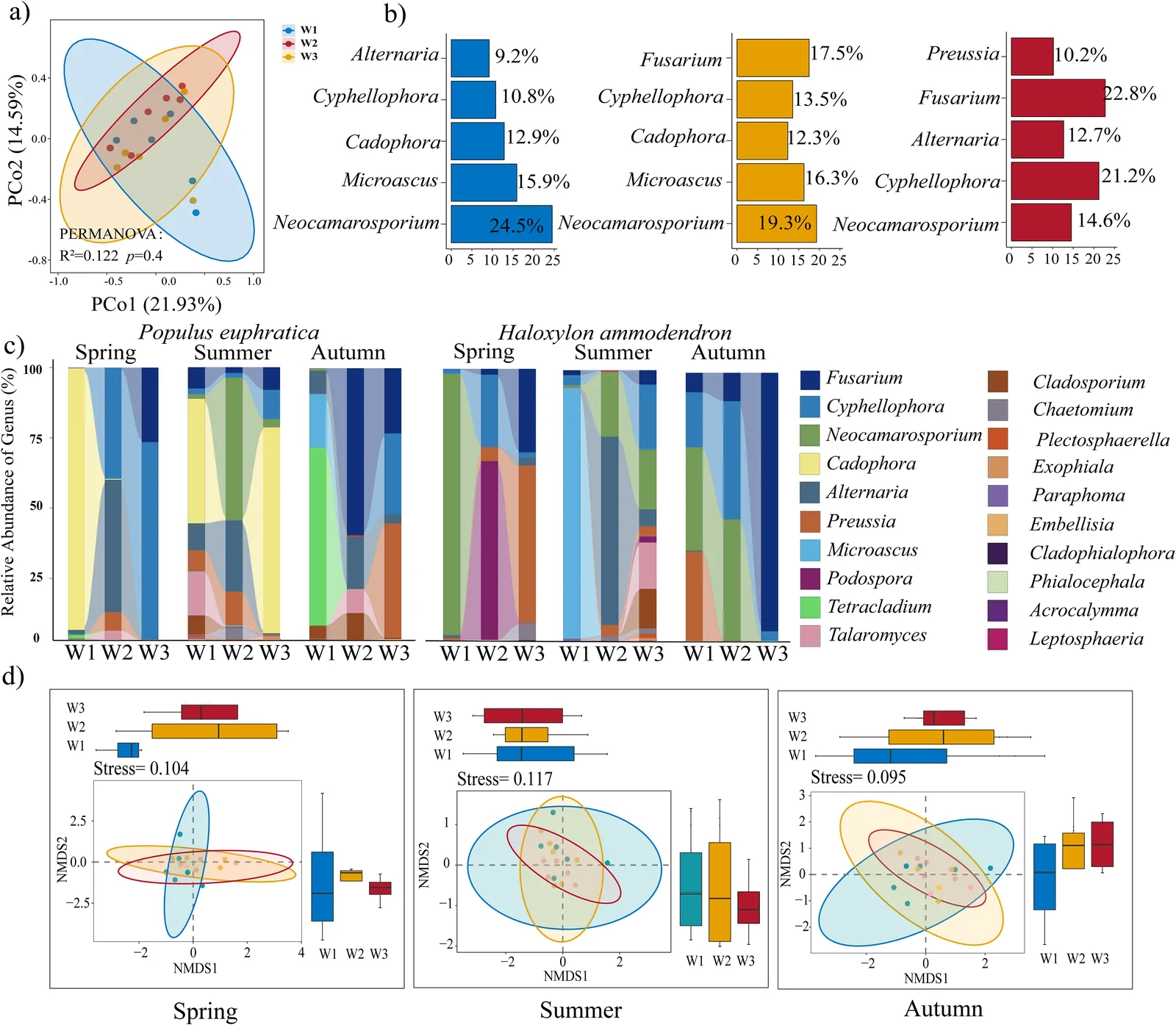
DSE community composition in the roots of *P. euphratica* and *H. ammodendron* under seasonal variation and different drought conditions. **(a)** Differences in DSE community distribution under different drought conditions based on Bray-Curtis dissimilarities calculated from the OTU absolute abundance matrix. **(b)** Major taxa contributing to DSE community dissimilarity based on SIMPER analysis. **(c)** Relative abundance-based DSE community composition across seasons, plant species, and drought conditions, showing proportional changes in dominant taxa. **(d)** Differences in DSE community composition among drought conditions within each season based on the OTU absolute abundance matrix. W1, W2, and W3 represent low, moderate, and high drought conditions, respectively.

Although the composition of the DSE community varied across seasons and drought conditions, drought conditions did not have a significant effect on the overall community structure (PERMANOVA: R^2^ = 0.122, *p* = 0.4; **Figure 6a**). SIMPER analysis indicated that community differences were primarily contributed by the genera *Neocamarosporium*, *Fusarium*, *Microascus*, and *Cyphellophora*. However, the Kruskal-Wallis test showed that these differences were not statistically significant (**Figure 6b**). In spring, the roots of *P. euphratica* were dominated by *Cadophora* and *Neocamarosporium* under the W1 condition, whereas *Cyphellophora* and *Fusarium* were dominant under the W2 and W3 conditions. In summer, *Microascus* became the dominant genus (50.9%), while the relative abundance of *Neocamarosporium* decreased to 8.8% (**Supplementary Table 3**). By autumn, *Fusarium* gradually became the dominant taxon in the roots of *P. euphratica* (W2) and *H. ammodendron* (W3). DSE communities did not exhibit significant differences among different drought conditions within each season (**Figure 6d**). Comparisons among seasons indicated that only the genus *Chaetomium* showed significant variation across seasons (*p* < 0.05), whereas no significant seasonal differences were observed for the other dominant genera (**Supplementary Figure 2**). Indicator species analysis (IndVal) revealed that *Neocamarosporium* (0.92), *Microascus* (0.93), and *Alternaria* (0.90) had relatively high indicator values in *H. ammodendron* (**Supplementary Table 5**), suggesting a strong association of these taxa with *H. ammodendron* roots. In addition, *Cadophora*, *Tetracladium*, and *Cladophialophora* were detected exclusively in *P. euphratica*, whereas *Podospora* was detected exclusively in *H. ammodendron* (**Supplementary Table 5**).

### 3.3 Potential drivers of colonization rates and community structures in AMF and DSE, and their correlations with dominant taxa

**Figure 7:**
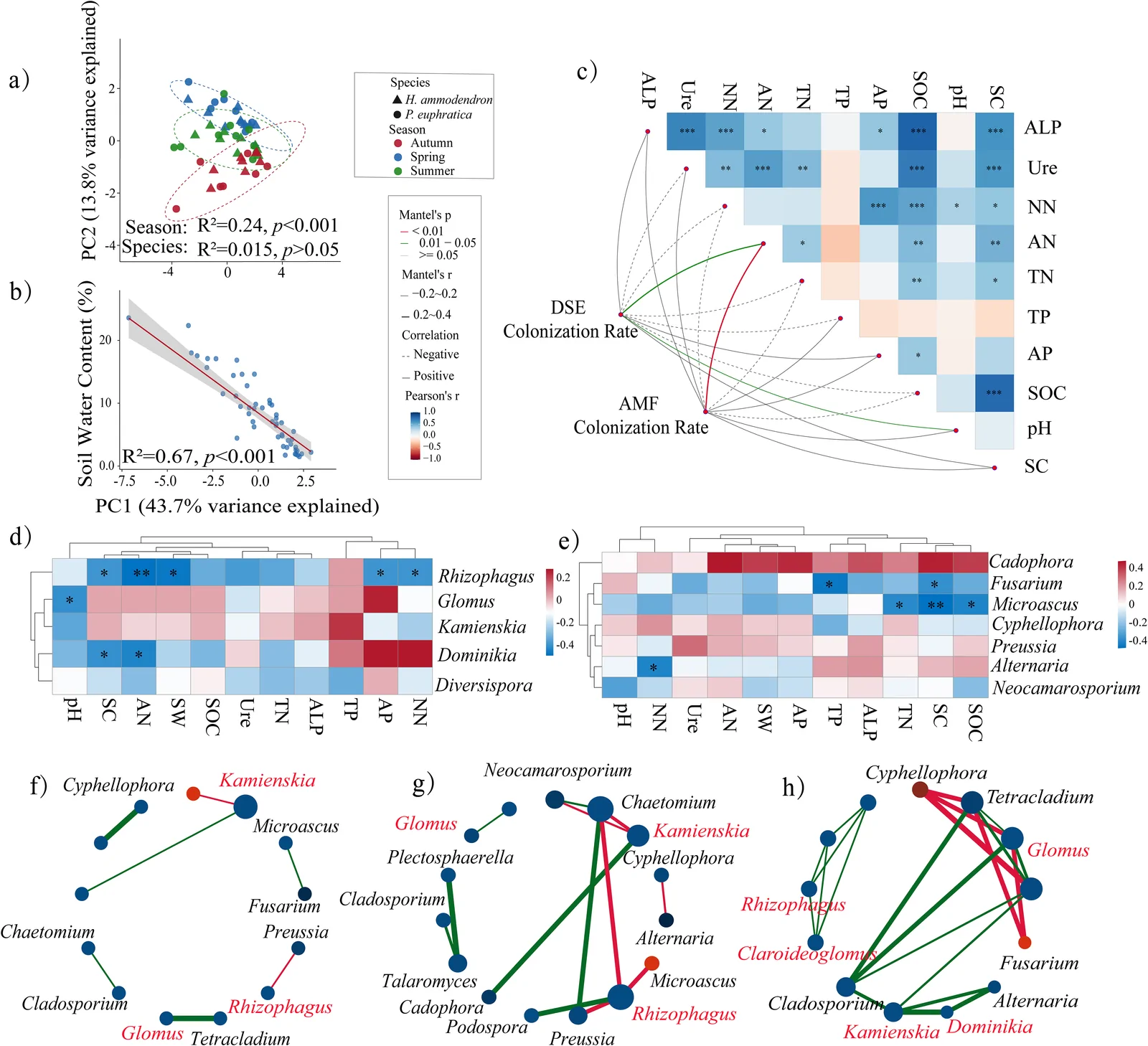
Potential drivers of AMF and DSE colonization rates, dominant taxa, and their co-occurrence patterns. **(a)** Principal component analysis (PCA) of soil environmental factors under different seasons and plant species. **(b)** Relationship between soil moisture content and principal component axes. **(c)** Mantel correlations between soil environmental factors and AMF and DSE colonization rates. **(d, e)** Correlation heatmaps showing associations between key taxa of the AMF **(d)** and DSE **(e)** communities and soil physicochemical factors. **(f–h)** Co-occurrence networks of dominant AMF and DSE genera in spring **(f)**, summer **(g)**, and autumn **(h)**. The co-occurrence networks were constructed based on genus-level Spearman correlations. AMF are indicated in red font and DSE in black font. Edge color represents correlation type (green for positive correlations and red for negative correlations), edge width indicates correlation strength, and node size and color represent taxon abundance. ***p < 0.001; **p < 0.01; *p < 0.05.

The physicochemical properties of rhizosphere soils associated with *P. euphratica* and *H. ammodendron* exhibited significant variation across seasons and drought conditions. With increasing drought intensity, soil moisture, salinity, soil organic carbon, nutrient contents (TP, AP, TN, AN, and NN), and enzyme activities (Ure and ALP) showed an overall decreasing trend (**Supplementary Figures 3**, **S4**). Principal component analysis revealed that PC1 and PC2 explained 43.7% and 13.8% of environmental variation, respectively, cumulatively accounting for 57.5% of total variance. Samples showed clear seasonal separation in PCA space, particularly along PC1, with autumn samples clustering on the negative side and spring and summer samples shifting toward the positive side (**Figure 7a**). Although some visual separation was apparent between samples associated with the two plant species, PERMANOVA indicated that plant species did not significantly explain overall environmental variation (R^2^ = 0.015, *p* > 0.05). In contrast, season explained a substantial proportion of environmental variation (R^2^ = 0.24, *p* < 0.001), indicating that seasonal variation was the primary source of soil environmental heterogeneity. PC1 scores were strongly negatively associated with soil water content (SW) (R^2^ = 0.67, *p* < 0.001; **Figure 7b**), consistent with the strong contribution of soil moisture to the first principal component. Multiple environmental variables demonstrated significant explanatory power in PCA space. Soil water content (SW; r^2^ = 0.767), soil organic carbon (SOC; r^2^ = 0.722), available phosphorus (AP; r^2^ = 0.719), and nitrate nitrogen (NN; r^2^ = 0.657) exhibited the strongest influences on sample distribution patterns (*p* < 0.001), indicating that these variables contributed strongly to environmental variation in the ordination space (**Supplementary Table 6**). Mantel tests indicated that both AMF and DSE colonization rates were significantly positively associated with variation in soil ammonium nitrogen (AN; *p* < 0.05) (**Figure 7c**). At the genus level, within the AMF community, *Rhizophagus* was significantly negatively correlated with SW, AN, AP, and NN; *Dominikia* was significantly negatively correlated with AN and SC; and *Glomus* was significantly negatively correlated with pH (*p* < 0.05) (**Figure 7d**). Within the DSE community, *Fusarium* showed significant negative correlations with SC and TP; *Microascus* was significantly negatively correlated with SOC, TN, and SC; and *Alternaria* was significantly negatively correlated with NN (*p* < 0.05) (**Figure 7e**).

The co-occurrence network structure of dominant AMF and DSE taxa exhibited pronounced seasonal variation (**Figures 7f–h**; **Supplementary Table 7**). From spring to autumn, network complexity increased progressively: the number of edges increased from 7 to 19 and 22; the average degree increased from 1.08 to 2.92 and 3.38; the clustering coefficient increased from 0 to 0.54 and 0.70; and network density increased from 0.09 to 0.24 and 0.28 (**Supplementary Table 7**). These results indicate that, with seasonal progression, inter-node connectivity gradually strengthened, community structure became more compact, and the network was predominantly characterized by positive correlations.

## 4 Discussion

Microscopic observations confirmed that AMF and DSE co-colonized the roots of *P. euphratica* and *H. ammodendron*. Their colonization structures and intensities varied across seasons, drought conditions, and host species, indicating context-dependent colonization responses to environmental variation. Trypan blue staining revealed that DSE hyphae and microsclerotia in *P. euphratica* appeared deep blue and brown, whereas in *H. ammodendron*, they were primarily brown (**Figure 3**). This variation may result from differences in chitin content in the hyphae and their binding affinities to the dye (Rozmoš et al., 2022). The microsclerotia in *P. euphratica* roots were compact and brain-shaped, while those in *H. ammodendron* were beaded and chain-shaped, suggesting that plant species influence the colonization structure of AMF and DSE (Liu et al., 2021). The results of this study indicate that drought significantly increased AMF colonization rates (**Figure 4a**), which is consistent with the findings of Augé (2001) and Staddon et al. (2003). Under drought conditions, the number of taxa within AMF communities often increases, particularly those belonging to the phylum Glomeromycota (de Vries et al., 2020). Similarly, in the present study, more AMF taxa were detected under high drought conditions, particularly in the roots of *P. euphratica* in autumn (**Figure 5c**; **Supplementary Table 2**). Previous studies have demonstrated that fungi within Glomeromycota can enhance host plant drought resistance by increasing antioxidant enzyme activity, alleviating oxidative stress, improving water-use efficiency, and promoting plant biomass accumulation (de Vries et al., 2020; Zuccarini et al., 2023). However, no AMF colonization was observed in the roots of *H. ammodendron* in autumn. This phenomenon may be associated with seasonal changes in environmental conditions. As day length shortens and temperature decreases, plant photosynthetic activity declines, thereby limiting the growth and colonization capacity of AMF (Dumbrell et al., 2011). The seasonal dynamics of AMF communities may be closely related to carbon resources allocated by the host plant to the rhizosphere. The availability of these carbon resources is jointly influenced by variations in day length, precipitation, and temperature across different growing seasons (Dumbrell et al., 2011; Zheng et al., 2014). DSE colonization was observed in plant roots under all three drought conditions (**Figure 4b**). This indicates that DSE exhibit lower sensitivity to environmental fluctuations (Jumpponen and Trappe, 1998; Huo et al., 2021) and are able to persist in host roots under diverse environmental conditions. Their community composition showed comparatively limited variation across seasons and drought conditions (**Figure 6**), suggesting that DSE may be less sensitive to environmental variation than AMF (Postma et al., 2007; Alberton et al., 2010). Furthermore, Netherway et al. (2024) reported that, compared with AMF, DSE form a closer association with plant roots, further indicating that these two fungal groups may perform distinct ecological functions within rhizosphere ecosystems.

The results of this study showed that drought conditions significantly affected AMF community composition, whereas DSE community composition remained comparatively stable across the three drought conditions (**Figures 5a**, **6a**). With increasing drought intensity, the AMF community exhibited pronounced differentiation, and these differences were primarily driven by the genera *Kamienskia*, *Glomus*, *Rhizophagus*, and *Diversispora* (**Figure 5b**). Among these, *Kamienskia*, *Glomus*, and *Rhizophagus* all belong to the family Glomeraceae, indicating that members of Glomeraceae contributed strongly to drought-associated differences in AMF community composition. Similar patterns have been observed in other desert ecosystems. For example, Xie et al. (2024a), in a study of *Echinops gmelinii* in the Tengger Desert, reported a significant increase (28.3%) in the abundance of drought-tolerant taxa within Glomeraceae under high-intensity drought conditions, indicating a pronounced response of AMF communities to drought. Furthermore, previous studies have demonstrated that *Glomus* can establish stable symbiotic associations with a wide range of plant species and significantly alleviate various abiotic and biotic stresses (Qiu et al., 2021; Karimi and Noori, 2022). For instance, Alipour et al. (2021) found that inoculation with *Glomus* significantly enhanced the growth performance of *Foeniculum vulgare* Mill under water stress conditions. Numerous studies have similarly demonstrated that AMF community structure is highly sensitive to drought variation (Kim et al., 2015; Pérez-Ramos et al., 2021; Alguacil et al., 2021; Tang et al., 2024). In the present study, *Septoglomus*, *Dominikia*, and *Diversispora* were detected only in *P. euphratica* (**Supplementary Table 4**), indicating a certain degree of host specificity. This finding is consistent with Grünfeld et al. (2021), who reported that AMF may exhibit pronounced host preference under certain conditions, although the strength of this preference is often jointly influenced by environmental heterogeneity, spatial scale, and local ecological processes. In contrast, *Kamienskia*, *Glomus*, and *Rhizophagus* were shared taxa between the two plant species (**Supplementary Table 4**), exhibiting lower host specificity and the ability to coexist with a broader range of plants. This characteristic may enable them to better adapt to highly fluctuating ecological conditions under drought (Zhou et al., 2018; Han et al., 2025). These results further indicate that AMF communities are highly responsive to drought conditions, with marked shifts in community composition as drought intensity increased.

In contrast to the pronounced response of AMF communities, DSE community composition remained comparatively stable across the three drought conditions. Both PCoA and PERMANOVA analyses indicated no significant differences in DSE community structure under different drought conditions (**Figure 6a**). Although SIMPER analysis suggested that genera such as *Neocamarosporium*, *Fusarium*, and *Microascus* contributed substantially to community dissimilarity, the results of the Kruskal-Wallis test were not statistically significant (**Figure 6**), further demonstrating the strong stability of the DSE community structure. In this study, *Neocamarosporium*, *Microascus*, and *Alternaria* maintained relatively high abundance and stable dominance in the root-associated fungal community of *H. ammodendron* (**Supplementary Table 5**; **Figure 6b**). Previous studies have shown that these genera are widely distributed in the rhizosphere of desert plants and can promote host plant growth under drought conditions, indicating their potential ecological supporting roles in arid ecosystems (Liu et al., 2023; He et al., 2022; Han et al., 2025). In addition, although most taxa contributing substantially to community dissimilarity were shared between the two plant species, *Cadophora*, *Tetracladium*, and *Cladophialophora* were predominantly detected in *P. euphratica* (**Supplementary Table 5**). Among them, *Cladophialophora* has been reported to regulate plant nutrient uptake, alleviate disease incidence, and promote plant growth (Harsonowati et al., 2020; Santos et al., 2021; Zhang et al., 2022). In contrast, *Podospora* was mainly detected in *H. ammodendron* (**Supplementary Table 5**), suggesting that although DSE lack strict host specificity, they may still exhibit a certain degree of host preference. This characteristic is consistent with the broad ecological adaptability of DSE. Previous studies have demonstrated that DSE are widely distributed in the roots of diverse plant species and across various soil environments (Kauppinen et al., 2013; Netherway et al., 2024), and can maintain relatively high colonization rates under drought or nutrient-poor conditions (Barrow, 2003; Mandyam and Jumpponen, 2005; Knapp et al., 2012). In addition, DSE can enhance host plant drought resistance by regulating plant physiological processes and interacting with other microorganisms (Li et al., 2019; Santos et al., 2021; Lu et al., 2025), and can significantly promote plant biomass accumulation under drought stress (Li et al., 2023).

Soil ammonium nitrogen content decreased overall with increasing drought intensity (**Supplementary Figure 3** and **S4**), while Mantel analysis indicated a significant positive association between AMF colonization and variation in soil ammonium nitrogen (**Figure 7c**). This pattern suggests that AMF colonization covaried with nitrogen availability across the sampled environmental conditions. Previous studies have shown that AMF can absorb ammonium through their hyphal networks and transfer nitrogen to host plants, providing a possible ecological context for the observed association (He et al., 2018; Püschel et al., 2023; Trombley et al., 2025; Ahmed et al., 2026). In addition, through their hyphal networks, AMF can enhance ammonium nitrogen availability in the rhizosphere, thereby further promoting plant nitrogen uptake (Trombley et al., 2025). Previous studies have shown that inoculation with AMF (*Rhizophagus intraradices*) significantly reduced soil ammonium nitrogen concentration (He et al., 2018). In the present study, a significant negative correlation was observed between *Rhizophagus* and ammonium nitrogen concentration (**Figure 7d**). Ahmed et al. (2026) further demonstrated that AMF efficiently absorb ammonium nitrogen through their hyphal networks and transfer nitrogen to plants via carbon-nitrogen exchange mechanisms, thereby enhancing plant nitrogen uptake efficiency. This phenomenon has been verified under a range of conditions from adequate water availability to severe drought (Püschel et al., 2023). Similarly, DSE colonization rates were significantly positively associated with variation in soil ammonium nitrogen (**Figure 7c**). Previous studies have demonstrated that DSE colonization can effectively enhance host plant nitrogen acquisition (Ruotsalainen et al., 2021). At the genus level, several dominant DSE taxa showed significant negative correlations with total nitrogen, total phosphorus, and soil organic carbon (**Figure 7e**), indicating that the distributions of these taxa were associated with soil carbon and nutrient availability. Previous studies have indicated that DSE can alter the quality of soil organic matter (Xu et al., 2020), and that the soil C/N ratio is a key factor regulating DSE colonization (Netherway et al., 2024). Currently, increasing attention has been directed toward the ecological functions of DSE in plant nutrient acquisition. DSE colonization can enhance plant nitrogen acquisition under inorganic nitrogen-deficient conditions (Newsham, 2011), promote soil carbon and nitrogen accumulation through the turnover of recalcitrant hyphal necromass (See et al., 2021), and increase plant nitrogen uptake under low-nitrogen conditions (Xu et al., 2020). Taken together, nitrogen availability plays an important role in regulating both AMF and DSE colonization. This finding is consistent with previous studies and further suggests that nitrogen availability is a key factor modulating endophytic fungal colonization and host plant growth (Johnson et al., 2014; Wang et al., 2018).

Co-occurrence network analysis revealed pronounced seasonal shifts in the association patterns between AMF and DSE. The spring network contained a relatively high proportion of negative correlations, suggesting contrasting abundance responses among dominant taxa. In contrast, the autumn network exhibited greater complexity and was dominated by positive correlations, indicating closer covariation among dominant taxa. These seasonal shifts may reflect shared environmental filtering and differences in the ecological roles and resource-use strategies of AMF and DSE. For example, AMF can enhance rhizosphere water acquisition through extensive hyphal networks, whereas DSE have been reported to improve water retention and plant drought tolerance (He et al., 2019). Co-inoculation studies have also reported positive effects of AMF and DSE on plant growth, nutrient acquisition, and stress tolerance, although these effects vary with fungal identity, inoculum density, host species, and environmental conditions (Xie et al., 2021; Liu et al., 2026). Field studies further indicate that AMF and DSE may respond independently to environmental gradients, and their associations may be shaped by differential environmental responses and links to distinct ecological processes (Huo et al., 2021; Netherway et al., 2024). Taken together, changes in fungal colonization and community composition across drought conditions, together with seasonal shifts in co-occurrence network complexity, provide field-based evidence for a seasonal reorganization of the association structure between AMF and DSE, offering a basis for future experimental evaluation of the ecological processes underlying these patterns.

However, the comparison of AMF and DSE ecological strategies should be interpreted with caution, because primer-related bias may exist in this study. Universal ITS primers can profile broad root-associated fungal communities, but they may underrepresent Glomeromycota due to limited taxonomic resolution and uneven amplification efficiency, whereas many DSE-related Ascomycota taxa are more readily captured by ITS-based sequencing (Öpik et al., 2013; Perez-Lamarque et al., 2022; Guillen-Otero et al., 2023). Thus, the apparently lower diversity or stronger drought sensitivity of AMF may partly reflect methodological under-detection, which could exaggerate the contrast between drought-sensitive AMF and relatively stable DSE strategies. Nevertheless, ITS-based sequencing can still reveal ecologically meaningful AMF community shifts within a consistent marker framework, and the observed ITS-based community changes provide valuable evidence for understanding the responses of AMF and DSE to drought conditions (Kohout et al., 2014; George et al., 2019; Guillen-Otero et al., 2023). Future studies combining ITS profiling with AMF-specific 18S or LSU rRNA markers are needed to better separate primer effects from true ecological differences between AMF and DSE.

## 5 Conclusion

This study examined how seasonal variation and drought conditions shape AMF and DSE colonization and community structure in the roots of *P. euphratica* and *H. ammodendron*. Drought strongly affected fungal colonization, particularly AMF colonization in *P. euphratica*, and these responses varied between host species. AMF communities changed markedly across drought conditions, whereas DSE communities remained comparatively stable, suggesting contrasting response patterns between the two fungal groups. Their co-occurrence structure also varied seasonally, with the autumn network showing the greatest complexity and a predominance of positive correlations, indicating a seasonal reorganization of the associations between AMF and DSE. Overall, these findings demonstrate that drought, seasonality, and host identity jointly shape the colonization, community composition, and association patterns of root endophytic fungi in desert plants. This study advances our understanding of belowground fungal responses to environmental stress and provides an ecological basis for dryland restoration. Future studies combining taxon-specific molecular markers, manipulative experiments, and functional analyses are needed to clarify the ecological processes underlying these patterns and their consequences for plant drought resistance.

## Data Availability

The datasets presented in this study can be found in online repositories. The names of the repository/repositories and accession number(s) can be found in the article/**Supplementary Material**.
