# Supplementary material for: Seasonal variation and drought effects on colonization and community structure of arbuscular mycorrhizal fungi and dark septate endophytes in desert plants
Source: Front Plant Sci. 2026 Sep 4;17:1849641. doi: 10.3389/fpls.2026.1849641 (PMC13586028; doi:10.3389/fpls.2026.1849641)
Supplement: Supplementary file 1 [file SupplementaryFile1.docx]

# **Table S1** Methods for determining soil physicochemical factors

| Soil Factors | Method |
| --- | --- |
| SW | Gravimetric method |
| SC | Electrical conductivity method |
| pH | pH meter method |
| SOC | Potassium dichromate oxidation-external heating method |
| TN | Kjeldahl method |
| TP | Molybdenum antimony colorimetric method |
| AP | Sodium bicarbonate extraction-molybdenum antimony colorimetric method |
| NN | Phenol disulfonic acid method |
| AN | Indophenol blue colorimetric method |
| Ure | Sodium phenolate-sodium hypochlorite colorimetric method |
| ALP | Disodium phenyl phosphate colorimetric method |

Note: Soil water content (SW), soil salinity (SC), soil pH (pH), soil organic carbon (SOC) content, total phosphorus (TP), total nitrogen (TN), available phosphorus (AP), ammonium nitrogen (AN), nitrate nitrogen (NN), urease (Ure), and alkaline phosphatase (ALP).

| **Phylum** |  | | | | |
| --- | --- | --- | --- | --- | --- |
| Glomeromycota |  |  |  |  |  |
| **Class** |  |  |  |  |  |
| Glomeromycetes |  |  |  |  |  |
| **Order** (2) | **Family** (3) | **Genus** (7) | Spring (%) | Summer (%) | Autumn (%) |
| Glomerales | Glomeraceae | *Dominikia* | 0.91 | —— | 7.56 |
|  |  | *Glomus* | 17.80 | 67.93 | 7.50 |
|  |  | *Kamienskia* | 49.48 | 9.49 | 16.76 |
|  |  | *Rhizophagus* | 6.75 | 3.76 | 1.54 |
|  |  | unclassified_o__Glomerales | 3.89 | —— | 0.64 |
|  |  | *Septoglomus* | —— | 0.05 | —— |
|  |  | unclassified_p__Glomeromycota | 4.81 | —— | 2.18 |
|  |  | unclassified_f__Glomeraceae | 14.42 | 15.20 | 61.73 |
|  | Claroideoglomeraceae | *Claroideoglomus* | 1.95 | 0.09 | 2.08 |
| Diversisporales | Diversisporaceae | *Diversispora* | —— | 3.48 | —— |

# **Table S2** Relative abundance and taxonomic classification of AMF genera across seasons

# **Table S3** Relative abundance and taxonomic classification of DSE genera across seasons

| **Phylum** (1) | **Class** (4) | **Order** (9) | **Family** (17) | **Genus** (20) | Spring (%) | Summer (%) | Autumn (%) |
| --- | --- | --- | --- | --- | --- | --- | --- |
| Ascomycota | Dothideomycetes | Pleosporales | Pleosporaceae | *Alternaria* | 0.92 | 18.76 | 1.43 |
|  |  |  |  | *Embellisia* | —— | —— | 0.05 |
|  |  |  |  | *Neocamarosporium* | 33.09 | 8.77 | 23.98 |
|  |  |  | Sporormiaceae | *Preussia* | 3.75 | 2.55 | 14.26 |
|  |  |  | Phaeosphaeriaceae | *Paraphoma* | 0.03 | 0.44 | —— |
|  |  |  | Leptosphaeriaceae | *Leptosphaeria* | —— | —— | <0.01 |
|  |  |  | Morosphaeriaceae | *Acrocalymma* | —— | —— | 0.01 |
|  |  | Capnodiales | Cladosporiaceae | *Cladosporium* | 0.02 | 0.93 | 0.51 |
|  | Eurotiomycetes | Chaetothyriales | Herpotrichiellaceae | *Cladophialophora* | —— | 0.02 | 0.01 |
|  |  |  |  | *Exophiala* | —— | 0.10 | 0.01 |
|  |  |  | Cyphellophoraceae | *Cyphellophora* | 28.57 | 3.33 | 24.56 |
|  |  | Eurotiales | Trichocomaceae | *Talaromyces* | 0.02 | 1.90 | 0.12 |
|  | Leotiomycetes | Helotiales | Helotiales_fam_Incertae_sedis | *Cadophora* | 5.35 | 8.87 | —— |
|  |  |  | Vibrisseaceae | *Phialocephala* | —— | 0.03 | —— |
|  |  |  | Helotiaceae | *Tetracladium* | 0.08 | —— | 3.68 |
|  | Sordariomycetes | Glomerellales | Plectosphaerellaceae | *Plectosphaerella* | —— | 0.16 | —— |
|  |  | Hypocreales | Nectriaceae | *Fusarium* | 9.33 | 2.42 | 30.18 |
|  |  | Microascales | Microascaceae | *Microascus* | 0.01 | 50.95 | 1.19 |
|  |  | Sordariales | Lasiosphaeriaceae | *Podospora* | 18.62 | 0.03 | —— |
|  |  |  | Chaetomiaceae | *Chaetomium* | 0.25 | 0.74 | 0.01 |

**Table S4** Integrated IndVal analysis results of AMF across different host plant communities

| Genus | *P. euphratica* | *H. ammodendron* | Index | IndVal | p-value |
| --- | --- | --- | --- | --- | --- |
| *Kamienskia* | 0.89 | 0.11 | 1 | 0.84 | 0.02 |
| unclassified_f__Glomeraceae | 0.97 | 0.03 | 1 | 0.71 | 0.04 |
| *Glomus* | 1.00 | 0.00 | 1 | 0.75 | 0.05 |
| *Rhizophagus* | 0.89 | 0.11 | 1 | 0.68 | 0.24 |
| *Diversispora* | 1.00 | 0.00 | 1 | 0.28 | 0.54 |
| *Claroideoglomus* | 0.96 | 0.04 | 1 | 0.44 | 0.29 |
| *Dominikia* | 1.00 | 0.00 | 1 | 0.57 | 0.06 |
| unclassified_p__Glomeromycota | 0.82 | 0.18 | 1 | 0.40 | 0.41 |
| unclassified_o__Glomerales | 0.60 | 0.40 | 1 | 0.35 | 0.75 |
| *Septoglomus* | 1.00 | 0.00 | 1 | 0.28 | 0.54 |

Note: Values under *P. euphratica* and *H. ammodendron* represent the normalized association of each taxon with the two host plant communities, with values closer to 1 indicating a stronger association with the corresponding host. Index indicates the host group with which each taxon showed the highest association (1 = *P. euphratica*; 2 = *H. ammodendron*). IndVal represents the indicator value (range: 0–1), with higher values indicating a stronger association between a taxon and the corresponding host community. p-value indicates the significance of the indicator taxon analysis; *p* < 0.05 was considered statistically significant.

**Table S5** IndVal analysis results of DSE across different host plant communities

| Genus | *P. euphratica* | *H. ammodendron* | | Index | IndVal | p-value |
| --- | --- | --- | --- | --- | --- | --- |
| *Neocamarosporium* | 0.04 | 0.96 | 2 | | 0.92 | 0.06 |
| *Fusarium* | 0.29 | 0.71 | 2 | | 0.84 | 0.36 |
| *Cyphellophora* | 0.43 | 0.57 | 2 | | 0.76 | 0.66 |
| *Microascus* | 0.03 | 0.97 | 2 | | 0.93 | 0.08 |
| *Preussia* | 0.39 | 0.61 | 2 | | 0.69 | 0.46 |
| *Alternaria* | 0.19 | 0.81 | 2 | | 0.90 | 0.99 |
| *Cadophora* | 1.00 | 0.00 | 1 | | 0.67 | 0.08 |
| *Podospora* | 0.00 | 1.00 | 2 | | 0.67 | 0.24 |
| *Tetracladium* | 1.00 | 0.00 | 1 | | 0.58 | 0.20 |
| *Talaromyces* | 0.90 | 0.10 | 1 | | 0.71 | 0.73 |
| *Cladosporium* | 0.86 | 0.14 | 1 | | 0.82 | 0.45 |
| *Chaetomium* | 0.28 | 0.72 | 2 | | 0.75 | 0.43 |
| *Plectosphaerella* | 0.64 | 0.36 | 1 | | 0.46 | 0.84 |
| *Exophiala* | 0.90 | 0.10 | 1 | | 0.63 | 0.18 |
| *Cladophialophora* | 1.00 | 0.00 | 1 | | 0.47 | 0.48 |

Note: Values under *P. euphratica* and *H. ammodendron* represent the normalized association of each taxon with the two host plant communities, with values closer to 1 indicating a stronger association with the corresponding host. Index indicates the host group with which each taxon showed the highest association (1 = *P. euphratica*; 2 = *H. ammodendron*). IndVal represents the indicator value (range: 0–1), with higher values indicating a stronger association between a taxon and the corresponding host community. p-value indicates the significance of the indicator taxon analysis; *p* < 0.05 was considered statistically significant.

# **Table S6** Results of environmental variable fitting (envfit) on PCA axes

| Variable | PC1 | PC2 | r^2^ | p-value |
| --- | --- | --- | --- | --- |
| SW | -0.8295 | 0.5585 | 0.7666 | 0.001 |
| SC | -0.94413 | -0.32957 | 0.6524 | 0.001 |
| pH | -0.11672 | 0.99316 | 0.5365 | 0.001 |
| SOC | -0.95017 | 0.31172 | 0.7215 | 0.001 |
| AP | -0.59123 | -0.80651 | 0.7193 | 0.001 |
| TP | 0.71486 | -0.69926 | 0.0659 | 0.100 |
| TN | -0.50893 | 0.8608 | 0.5342 | 0.001 |
| AN | -0.97308 | 0.23048 | 0.3701 | 0.001 |
| NN | -0.77039 | -0.63757 | 0.6566 | 0.001 |
| Ure | -0.89695 | 0.44212 | 0.6549 | 0.001 |
| ALP | -0.70538 | -0.70883 | 0.6532 | 0.001 |

Note: Results of the envfit() analysis projecting environmental variables onto the PCA ordination space. PC1 and PC2 represent the projection coordinates of each environmental variable, r^2^ indicates the strength of the association between each variable and the ordination configuration, and p-values were obtained from 999 permutations.

# **Table S7** Topological properties of seasonal co-occurrence networks among dominant AMF and DSE genera

| Network complexity indicators | Spring | Summer | Autumn |
| --- | --- | --- | --- |
| Number of nodes | 13 | 13 | 13 |
| Number of edges | 7 | 19 | 22 |
| Average degree | 1.08 | 2.92 | 3.38 |
| Clustering coefficient | 0 | 0.54 | 0.70 |
| Network density | 0.09 | 0.24 | 0.28 |
| Positive correlation ratio | 0.71 | 0.79 | 0.77 |
| Negative correlation ratio | 0.29 | 0.21 | 0.23 |

# **Figure S1.** Pairwise seasonal comparisons of AMF community composition and identification of key indicator taxa

Note: Indicator taxon analysis was performed using pairwise comparisons among seasons to identify taxa driving seasonal differences in AMF community composition. IndVal (0–1) represents the strength of taxon association with a given season, with higher values indicating stronger and more consistent occurrence. Significance was assessed by permutation tests; *p* < 0.05 indicates a significant indicator taxon.

# **Figure S2.** Pairwise seasonal comparisons of DSE community composition and identification of key indicator taxa

# **Figure S3.** Seasonal variation of physicochemical factors in the rhizosphere soil of *P. euphratica* under different drought conditions. ****p* < 0.001; ** *p* < 0.01; * *p* < 0.05.

# **Figure S4.** Seasonal variation of physicochemical factors in the rhizosphere soil of *H. ammodendron* under different drought conditions. ****p* < 0.001; ** *p* < 0.01; * *p* < 0.05.
